# Nonfluent/Agrammatic PPA with In-Vivo Cortical Amyloidosis and Pick’s Disease Pathology

**DOI:** 10.3233/BEN-2012-120255

**Published:** 2013

**Authors:** Francesca Caso, Benno Gesierich, Maya Henry, Manu Sidhu, Amanda LaMarre, Miranda Babiak, Bruce L. Miller, Gil D. Rabinovici, Eric J. Huang, Giuseppe Magnani, Massimo Filippi, Giancarlo Comi, William W. Seeley, Maria Luisa Gorno-Tempini

**Affiliations:** ^1^Memory and Aging CenterUniversity of CaliforniaSan FranciscoCAUSA; ^2^Department of NeurologyScientific Institute and University Hospital San RaffaeleMilanItaly

**Keywords:** Nonfluent primary progressive aphasia, PPA, apraxia of speech, Voxel-based morphometry, PiB-PET, Pick's disease, Alzheimer disease, Frontotemporal dementia

## Abstract

The role of biomarkers in predicting pathological findings in the frontotemporal dementia (FTD) clinical spectrum disorders is still being explored. We present comprehensive, prospective longitudinal data for a 66 year old, right-handed female who met current criteria for the nonfluent/agrammatic variant of primary progressive aphasia (nfvPPA). She first presented with a 3-year history of progressive speech and language impairment mainly characterized by severe apraxia of speech. Neuropsychological and general motor functions remained relatively spared throughout the clinical course. Voxel-based morphometry (VBM) showed selective cortical atrophy of the left posterior inferior frontal gyrus (IFG) and underlying insula that worsened over time, extending along the left premotor strip. Five years after her first evaluation, she developed mild memory impairment and underwent PET-FDG and PiB scans that showed left frontal hypometabolism and cortical amyloidosis. Three years later (11 years from first symptom), post-mortem histopathological evaluation revealed Pick's disease, with severe degeneration of left IFG, mid-insula, and precentral gyrus. Alzheimer’s disease (AD) (CERAD frequent/Braak Stage V) was also detected. This patient demonstrates that biomarkers indicating brain amyloidosis should not be considered conclusive evidence that AD pathology accounts for a typical FTD clinical/anatomical syndrome.

## 1. Introduction

The nonfluent/agrammatic variant of primary progressive aphasia (nfvPPA) is one of the three subtypes of PPA [1,2] for which consensus clinical diagnostic criteria have recently been updated [3]. NfvPPA is characterized by agrammatism and/or effortful, halting speech often consistent with apraxia of speech (AOS) and dysarthria [4]. Agrammatism causes oversimplification of language production, with lack of function words, inflections, and complex grammatical constructions. AOS is a motor speech disorder characterized by slow rate of speech, abnormal prosody, distorted sound substitutions, additions, repetitions and prolongations, sometimes accompanied by groping and trial-and error articulatory movements [4,5]. Patients with nfvPPA may have difficulty with comprehension of sentences that are syntactically complex but show preservation of single word comprehension.

ISSN 0953-4180/13/$27.50 © 2013 – IOS Press and the authors. All rights reserved

Neuroimaging studies have revealed that nfvPPA is anatomically associated with damage in the left posterior frontal gyrus (IFG), insula, and premotor/supplementary motor areas, as well as primary motor cortex [6–8].

NfvPPA is part of the frontotemporal dementia (FTD) clinical spectrum of disorders, which are most often caused by FTLD-type pathology such as microtubule-associated protein (tau) [9] and transactive response DNA binding protein of 43 kD (TDP-43) [10]. A recent meta-analysis [11] revealed that the diagnosis of nfvPPA is most commonly associated with FTLD-tau pathology (70%) that was split into three subtypes: Pick's disease (PiD), corticobasal degeneration (CBD) and progressive supranuclear palsy (PSP). NfvPPA with Alzheimer's disease (AD) pathology has also been reported [12–14], however certain clinical series [15, 16] report FTLD with TDP-43-immunoreactive inclusions (FTLD-TDP) as the most common finding. It has been proposed that, in the presence of a nfvPPA syndrome, motor speech impairment could predict CBD or PSP pathology [17], while a greater deficit in grammatical function would be associated with FTLD-TDP [18].

A novel PET ligand carbon11-labeled Pittsburgh compound-B ([11C] PiB) was recently introduced [19]. PiB binds specifically to fibrillar beta-amyloid (A$\beta$) so PiB-PET enables in vivo detection of cortical amyloidosis. The two studies that have applied this technique to PPA [20,21] reported results consistent with previous pathological data in showing that cortical amyloidosis is uncommon in nfvPPA as currently defined, while common in the logopenic variant (lvPPA). The question remains regarding whether the finding of a positive PiB scan in typical nfvPPA (or in other classic FTD syndromes) indicates sole causative AD pathology with an atypical anatomical pattern of neurodegeneration, co-occurrence of contributory FTLD and AD pathology with both contributing to cognitive symptoms, or an incidental finding of co-morbid amyloid plaques in a dementia driven by FTLD pathology. To our knowledge, no study has been conducted in nfvPPA in which in-vivo molecular neuroimaging findings were correlated with pathological diagnosis in the same patient.

Here we present a detailed, prospective, longitudinal clinical and neuroimaging study of FC (fictitious name), a 66 year old, right-handed woman with the typical features of a mainly speech-impaired nfvPPA who unexpectedly showed a PET-PIB positive scan and was found to have Pick's disease and AD pathological changes post-mortem. We present seven years of clinical and cognitive findings and five years of longitudinal structural MRI data. We argue that Pick's disease was the main cause of her clinical syndrome, while AD might have contributed to the development of mild late-emerging memory deficits.

### 1.1. Clinical report and methods

In 2003 (year 1), FC was seen at the University of California at San Francisco (UCSF) Memory and Aging Center. She gave written informed consent, and the study was approved by the Committee on Human Research at UCSF. FC received a comprehensive multidisciplinary evaluation including clinical history, general and neurological examination, neuropsychological testing, and neuroimaging. At that time, she was classified as having progressive non-fluent aphasia as described in the Gorno-Tempini et al. 2004 study [2]. She was included in the Rabinovici et al. PET_PiB PPA case series [22]. At that time, she would have also met a diagnosis of probable nfvPPA according to the current classification [3]. We followed the patient for seven years until death and autopsy was performed at the UCSF's Alzheimer's Disease Research Center.

### 1.2. Cognitive testing

The patient underwent a neuropsychological battery and a detailed speech and language evaluation during screening and follow-up visits. General intellectual function was assessed using the UCSF screening battery described elsewhere [23]. Due to FC's prominent language output deficits, written responses were allowed for many neuropsychological tests [e.g., The California Verbal Learning Test–Mental Status Version (CVLT-MS), backward digit span, semantic and phonemic fluency, and the abbreviated Boston Naming Test (BNT)]. However, she provided verbal responses to The Mini Mental Status Exam (MMSE) at year 1.

Motor speech was tested using the Motor Speech Evaluation (MSE) [24]. Spontaneous language production and single word-comprehension were evaluated using the "Spontaneous Speech," "Repetition" and "Auditory Word Recognition" subtests of the Western Aphasia Battery (WAB) [25]. Confrontation naming was evaluated using the 15-item version of the Boston Naming Test (BNT). To test visual semantic abilities, the three-picture version of the Pyramids and Palm Trees Test was administered [26]. Syntactic comprehension was tested using the WAB "Sequential Commands" and, more extensively, by selected subtests of the Curtiss–Yamada Comprehensive Language Evaluation–Receptive (CYCLE-R), [27]. The language evaluations were performed by a licensed speech and language pathologist.

The patient's raw scores on cognitive tasks were transformed into standardized z scores by using the mean and standard deviation from age-matched, normal control subjects who have participated in other studies at our center [n = 10, 5 male; mean age: 69.5 years (SD = 5.4; 5 men)] as published previously [2].

### 1.3. Neuroimaging protocol

#### 1.3.1. MRI scan

The patient underwent high resolution structural MRI scans on a 1.5-T Magnetom VISION system (Siemens Inc., Iselin, NJ) at the San Francisco Veterans Administration Medical Center [28]. A total of five annual MRI scans were collected starting at time of diagnosis and ending three years prior to death. Each image was obtained within three months of the clinical and cognitive evaluations.

All image processing and analysis were performed using SPM8 (http://www.?l.ion.ucl.ac.uk/spm/software/spm8) [29] and Matlab version 7.10 (The MathWorks, Inc.). All T1 structural images were segmented, bias-corrected and spatially normalized to Montreal Neurological Institute (MNI) space using a unified segmentation procedure [30]. The VBM analysis was conducted using modulated grey matter images, with voxel values multiplied by Jacobian determinants derived from the spatial normalization in order to preserve the total amount of grey matter from the original images. Modulated grey matter images were smoothed with a Gaussian kernel (12 mm FWHM). Each scan obtained from FC was compared to a control group comprising seventeen healthy right-handed females with a mean age of 67.7 (SD 2.99). None of the control subjects had any history of neurological or psychiatric disorders, and MRI scans were read as normal. Regionally-specific differences in gray matter volumes were assessed fitting a general linear model at each voxel. Age and total intracranial volume (TIV) were entered into the design matrix as nuisance variables. TIV was calculated by summing across the grey matter, white matter, and cerebrospinal fluid images, all modulated. The resulting statistical parametric map (SPM) was thresholded voxel-wise at $p < 0.01$, uncorrected, to avoid false-negatives that can occur in single-subjects VBM analyses.

#### 1.3.2. PET imaging

In 2007 the patient underwent positron emission tomography (PET) with the beta-amyloid ligand Pittsburgh Compound B (PiB) and with fluorodeoxyglucose (FDG). Image acquisition and analysis were performed as previously described [31]. For PiB, voxel-wise distribution volume ratio images were created (Logan graphical analysis, cerebellar reference), while FDG frames were normalized to mean activity in the pons.

#### 1.3.3. Genetic methods

Genetic analysis for *APOE* genotype was available such as for MAPT haplotype. APOE $\varepsilon 4$ is a well-known risk factor for AD [32] whereas the microtubule-associated protein $\tau$ (MAPT) H1/H1 allele was associated with tauopathies, such as (CBD) and PSP [33]. Genetic methods have been previously described [34].

#### 1.3.4. Neuropathology

The fresh brain was removed 8.5 hours post-mortem and cut into 8–10 mm-thick coronal slabs. These slabs were alternately fixed, in 10% neutral buffered formalin for 72 h, or rapidly frozen. Tissue blocks covering dementia-related left hemisphere regions of interest were dissected from the fixed slabs and basic and immunohistochemical stains were applied following standard diagnostic procedures developed for patients with dementia [35]. Immunohistochemistry was performed using antibodies to: TDP43 (anti-rabbit, 1:2000, Proteintech Group, Chicago, IL, USA), phosphorylated tau (CP-13 antibody, anti-mouse, 1:250, courtesy of P. Davies), phosphorylated 3R and4R tau (anti-mouse, 1:500, Millipore, Billericia, MA, USA), beta-amyloid (anti-mouse, 1:250, Millipore, Billerica, MA, USA), alpha synuclein (anti-mouse, 1:1000, Millipore, Billerica, MA, USA). All immunohistochemical runs included positive control sections to exclude technical factors as a cause of absent immunoreactivity.

### 1.4. Clinical report

#### 1.4.1. First evaluation

At her first visit to UCSF (year 1) FC was a 66 year-old, a right-handed woman with a three-year history of speech and language difficulties that were gradually worsening over time. She was born in the US but raised in a family where she learned Spanish as her first language. At age five she started attending an English-speaking school. She remained bilingual throughout her life and spoke English outside the home and Spanish with her family. She worked for several years as a radiology technician and later as a teacher in a daycare center. She stopped working at age 63, when her language symptoms became apparent.

She reported that she first noticed "stuttering" and having difficulties pronouncing words that she nevertheless had "in her mind". Initially, she could still pronounce most words correctly but more slowly and with greater effort. With time, it became more difficult for her to produce certain words and she would make errors in pronunciation. She also reported that she had become slow in putting together sentences and that she would say words "out of order". These difficulties slowly progressed over the three years prior to her first UCSF visit to the point where she had significant problems producing most words and she preferred to communicate by writing. She reported that she was also slow in writing, with a tendency to "stumble over words" and to make grammatical errors. Spanish and English were equally affected. No general motor, memory, visuo-spatial, behavioral or executive difficulties were reported and her activities of daily living were limited only by difficulties with verbal communication.

She first saw her primary care doctor in 2002 and then a neurologist and a psychiatrist who were concerned about a stroke and obtained an MRI of FC's brain. The MRI failed to reveal a stroke and the patient sought a second opinion with another neurologist in the same year. The second neurologist observed problems in word-finding, slow speech and some difficulties in reading and writing. On that occasion the patient underwent B12 dosage, routine lumbar puncture and a FDG-PET scan. Despite normal findings on these studies by report, the neurologist explained to her that the speech difficulties were likely to be a neurodegenerative condition and she was referred to UCSF.

In 2003 (year 1) Mrs. FC was evaluated at the UCSF Memory and Aging Center.

Past medical history was significant only for mild diet-controlled hypercholesterolemia and her medications included a multivitamin and calcium supplementation daily. There was no history of neurologic/neurodegenerative disease in FC's family.

General physical examination was unremarkable. On neurological examination, FC was alert, oriented and cooperative. Her speech was effortful and markedly slowed. She had difficulty producing simple syllables such as "pa", "ta", "ka" and producing the names of simple objects such as "pen". Repetition was severely impaired due to motor speech difficulties. She was unable to sustain a vowel. Her cranial nerve examination was unremarkable except for the presence of saccadic smooth pursuit and increased gag reflex. On motor examination, she showed mild decrease of motor dexterity in the right hand. Her tone was mildly increased in the right greater than left upper extremities. Gait was notable for mildly decreased arm swing bilaterally with unstable tandem gait. Deep tendon reflexes were brisk in all limbs but symmetrical. Plantar responses were flexor bilaterally.

Neuropsychological screening revealed severe expressive difficulties and written responses were allowed in many tests that were thus scored in an unconventional way (for details see Table 1). She showed some difficulties in executive tests while memory and visuospatial abilities were unimpaired. Mini-mental state exam (MMSE) at this point did not allow for written responses and she therefore lost points for repetition of single words, repetition of the sentence and naming of the country, obtaining a score of 25/30.

On language testing, the patient showed greatest impairment on the WAB fluency measure, where she was able to produce only single words. In two minutes, she produced three intelligible words in response to the picnic picture. Overall intelligibility was approximately 12% on this task. The predominant cause of dysfluency was significant motor speech impairment, however written description of the picture was also limited. Writing was agrammatic but words were spelled correctly. Written picture description in four minutes was as follows: "The one afternoon the neighbor children is having a picnic front your house while the boy flying kite and other playing in water." The MSE revealed severe AOS and mild dysarthria with hyper-nasal resonance. Tongue movements were particularly impaired. Speech was characterized by groping, sound distortions and frequent pauses with increased intra- and intersegment duration. Varying degrees of morphological and syntactic errors were present in speech production, repetition, and passage reading tasks. On the WAB repetition task, the repetition of sentences was impaired, while single word repetition was relatively spared but distorted. FC scored 9/15 on the written BNT, with mainly articulatory errors. Semantic memory was relatively spared as indicated by performance on a semantic association task (Pyramid and palm trees pictures). Single word comprehension was within normal limits on the WAB subtest. On the more comprehensive CYCLE test, she made several errors on comprehension of the negative passives and on the object relatives with relativized objects. She showed mild bucco-facial apraxia, especially when movements of the tongue and lips were required.

Table 1

Demographic and cognitive assessment of the patient vs. control group

| BA | Year 1 | | | | Year 2 | | | | Year 3 | | | | Year 4 | | | | Year 5 | | | |
|---|---|---|---|---|---|---|---|---|---|---|---|---|---|---|---|---|---|---|---|---|
| | coordinates | Cluster p (unk) | Peak T | Extend (mm$^3$) | coordinates | Cluster p (unk) | Peak T | Extend (mm$^3$) | coordinates | Cluster p (unk) | Peak T | Extend (mm$^3$) | coordinates | Cluster p (unk) | Peak T | Extend (mm$^3$) | coordinates | Cluster p (unk) | Peak T | Extend (mm$^3$) |
| L SFG | −28, −4, 58 | 0.228 | 4.06 | 2912 | −26, −4, 58 | 0.431 | 3.9 | 1088 | −26, −4, 58 | 0.263 | 3.47 | 1328 | −26, −4, 58 | 0.101 | 4.55 | 2984 | −26, −4, 58 | 0,001* | 4,59 | 16784 |
| L MFG | – | – | – | – | – | . | . | . | – | – | – | – | −42, 6, 52 | 0.101 | 3.9 | 2984 | −42, 6, 52 | | 4.25 | |
| L Prec.G | −60, −2, 34 | 0.085 | 3.39 | 3328 | −38, 2, 54 | 0.431 | 3.04 | 664 | −40, 2, 52 | 0.263 | 3.39 | 1328 | −60, 0, 34 | 0,049* | 3,67 | 4512 | −58, 0, 36 | | 4.09 | |
| L PostcG. | −64, −6, 26 | | 3.25 | | −64, −6, 26 | 0.145 | 3.03 | 2312 | −62, −2, 34 | 0.195 | 3.07 | 1800 | −62, −2, 34 | | 3.62 | | −62, −2, 34 | | 3.83 | |
| L IFG p.o. | −64, 2, 12 | | 3.04 | | −64, 2, 12 | | 2.98 | | −62, 2, 14 | | 3.14 | | −60, 2, 14 | | 3.5 | | −42, 12, 30 | | 4.36 | |
| L insula | −32, 14, 10 | 0.292 | 3.3 | 1176 | −32, 16, 10 | 0.576 | 2.97 | 352 | −32, 16, 10 | 0.362 | 3.26 | 880 | −32, 16, 8 | 0.409 | 3.48 | 728 | −34, 14, 8 | | 3.42 | |
| R Prec. G | 24, −12,66 | 0.228 | 4.16 | 1544 | 64, 6, 30 | 0.31 | 3.9 | 1088 | 64, 6, 30 | 0.586 | 3.28 | 336 | 64, 6, 30 | 0.197 | 4.03 | 1784 | 24, −12, 66 | 0.462 | 3.53 | 584 |
| R SMA | 4, 4, 70 | 0.519 | 3.71 | 456 | 4, 4, 70 | 0.672 | 3.23 | 216 | 4, 4, 68 | 0,367 | 4,01 | 864 | 4, 4, 68 | 0.237 | 4.44 | 1488 | 4, 4, 66 | 0.129 | 4.63 | 2520 |
| L caudate | −16, 18, 6 | 0.44 | 3.41 | 640 | −14, 18, 4 | 0.895 | 2.7 | 108 | −14, 16, 6 | 0.425 | 3.91 | 680 | −12, 16, 4 | 0.341 | 3.81 | 960 | −14, 16, 6 | 0.256 | 4.31 | 1368 |
| R caudate | – | – | – | – | – | – | – | – | – | – | – | – | 12, 18, 0 | 0.523 | 3.72 | 448 | 12, 18, 0 | 0.508 | 3.98 | 480 |
| L Hipp | – | – | – | – | – | – | – | – | – | – | – | – | – | – | – | – | −36, −18, −16 | 0.307 | 3.59 | 1104 |
| R Hipp | – | – | – | – | 32, −32, −2 | 0.755 | 2.91 | 128 | 32, −32, −2 | 0.932 | 2.66 | 16 | 30, −34, 0 | 0.738 | 2.83 | 144 | 32, −32, −4 | 0.393 | 3.49 | 776 |
| L Amyg. | – | – | – | – | – | – | – | – | – | – | – | – | – | – | – | – | −20, −2, −10 | 0.692 | 3.08 | 192 |

*Below Average; **Mildly impaired, ***Impaired patient's performance versus controls (based on Z score comparison);

NA: not applicable; MMSE. Mini-Mental State Examination; CDR: Clinical Dementia Rating; GDS: Geriatric Depression Scale; CVLT-MS: California Verbal Learning Test–Mental Status; VOSP: The Visual Object and Space Perception Battery; MSE: Motor Speech Evaluation.

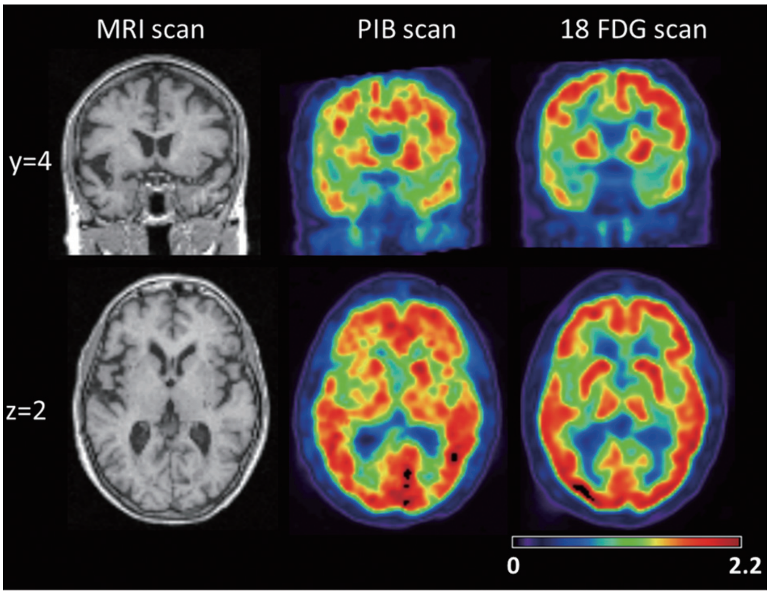

Fig. 1. PIB and FDG-PET obtained at Year 5. PET data were co-registered to the subject's most proximate T1-weighted MRI and are displayed in neurologic orientation.

The patient underwent brain MRI that, on visual examination, showed left greater than right insular atrophy and posterior inferior frontal atrophy. The presence of mild periventricular white matter T2 hyperintensities was noted, perhaps related to chronic subcortical vascular disease. APOE genotype was e3/e3 and tau haplotype H1/H1. She had no mutations in GRN, however MAPT was not sequenced.

In accordance with clinical-neuropsychological data and MRI findings, we made the consensus diagnosis of nfvPPA and hypothesized that the most probable underlying pathology was FTLD-tau and most likely CBD, although general motor impairment was very mild.

### *1.4.2. Progression*

In the following years, FC showed further impairment of spontaneous speech, becoming functionally mute. Her AOS and buccofacial apraxia worsened (with inability to generate cough, protrude the tongue etc.). At year 4, she could not repeat even single sounds. Semantic knowledge and naming were relatively preserved, as were the comprehension of single words and commands. No significant changes in other cognitive domains or functional abilities were observed, including cooking and house cleaning, nor were significant changes noted on formal neuropsychological testing. Her mild right extrapyramidal signs remained stable and not functionally disabling (she continued to dance with her husband). During this time the patient showed a persistent, significant weight loss initially related to a diet but persisting after the diet was stopped. At year 4 she performed an electromyography study that was normal, without any sign of motor neuron involvement. In order to exclude a neoplastic disorder the patient also underwent a chest x-ray and a gynecological exam that were normal. We suggested completing the screening with a colonoscopy and a mammogram but the patient refused them.

Visual evaluation of annual MRI brain scans from year 1 to year 4 showed a progression of the bi-insular atrophy (left significantly greater than right).

Beginning at year 5 (eight years from disease's onset), she developed questionable (because of severe language deficits) memory and executive impairments without significant functional changes. Her writing skills worsened and she displayed mild difficulties in comprehension of complex sentences. General motor examination remained stable. At this point she was still driving with no accidents reported. At that time, the patient's written MMSE score was 23/30 and her performance on neuropsychological testing showed mild decline in all domains, with the exception of visuospatial function. On language examinations she still showed very good comprehension skills, especially for single words, while sentence comprehension skills slowly declined. A follow-up MRI of the brain showed progression of left frontal atrophy and also mild bilateral hippocampal atrophy.

This year the patient underwent PiB and FDG-PET. As expected, FDG-PET revealed asymmetric left frontal glucose hypometabolism, especially in the insula and frontal operculum, whereas PiB scan showed diffuse cortical tracer binding in both hemispheres indicating fibrillar A$\beta$ deposition (Fig. 1).

At year 6, FC scored 17/30 in the MMSE, with written responses. She had difficulty naming objects and made spelling errors when writing. She was mute and unable to perform any verbal production task. She demonstrated comprehension difficulties, yes/no confusion, and severe bucco-facial and moderate limb apraxia. She was still able to recognize people and did not get lost in familiar environments. Her verbal and non-verbal memory test scores were below average, while visuospatial skills remained relatively intact.

During the following two years, FC experienced a general cognitive decline but continued to participate in family gatherings. She followed her favorite television programs and was able to move well, remaining independent in eating, walking and all her ADLs until the last months of her disease.

Beginning in year 3, FC reported progressive swallowing difficulties and, during year 8, episodes of dysphagia increased in frequency and severity.

### *1.5. Neuroimaging- VBM analysis*

The comparison of FC's image from year 1 (Table 2, Fig. 2) versus controls revealed decreased grey matter volume in the left dorsal anterior insula, pars opercularis of the IFG, caudate, bilateral precentral gyrus and right supplementary motor area. VBM findings showed the same general pattern of atrophy for scans obtained in year 2 and year 3. At year 4, the same cortical regions showed further involvement (Table 2), particularly the pars opercularis of the IFG and precentral gyrus. In the scan obtained at year 5, parts of the left premotor cortex, including superior and middle frontal gyrus, and pars opercularis of the inferior frontal gyrus showed further decrease in grey matter volume, reaching $p < 0.01$ at cluster level. Furthermore, bilateral supplementary motor area, caudate (left > right), hippocampi and left amygdala showed decreased volume.

### *1.6. Neuropathology*

The patient developed aspiration pneumonia and died from its complications 8 years from first evaluation and 11 years from first symptom. Her husband provided informed consent for the patient to undergo brain autopsy. The fresh brain weighed 1053 grams, and gross examination revealed severe focal atrophy involving the left inferior frontal gyrus (pars opercularis), dorsal anterior insula, and precentral gyrus. Nonspecific neurodegenerative changes, including microvacuolation, gliosis, and neuronal loss were most severe in the left opercular IFG; the precentral gyrus, in the vicinity of the face, mouth, and pharyngeal motor representations; and the middle insula. Immunohistochemical analysis revealed Pick's disease (Fig. 3), with frequent Pick bodies in inferior frontal gyrus (pars opercularis), precentral gyrus, middle insula, middle frontal gyrus, anterior cingulate cortex, striatum, claustrum, dentate gyrus, and CA1/subiculum. Furthermore, there were diffuse/granular neuronal cytoplasmic inclusions, dystrophic tau-positive astrocytes, and copious neuropil threads, all consistent with the diagnosis of Pick's disease. In addition, there was a significant burden of Alzheimer's disease-related pathology, with abundant neuritic plaques and neurofibrillary tangles in neocortex consistent with CERAD frequent/Braak Stage V (NIA-Reagan high-likelihood AD) and moderate amyloid angiopathy.

## 2. Discussion

We report comprehensive, prospective longitudinal clinical and neuroimaging data for a patient with typical nfvPPA [3] who was PiB+ and who was found to have Pick's disease and AD at autopsy. We discuss her neuroimaging and clinical features and argue that positivity of AD biomarkers should not exclude the presence of FTLD pathology in patients with a typical nfvPPA clinical syndrome.

In FC, motor speech impairment was so severe that soon after her first presentation to our clinic she became functionally mute. She also showed mild agrammatism (verbal and written initially and written only later) but it was mild. Detailed evaluation of FC's motor speech impairment was performed only at year one, when she was still able to produce some words. AOS was characterized by effortful groping, slow rate, sequencing errors, sound distortions and dysprosody. Dysarthria was mixed and difficult to classify. FC therefore showed a pattern of impairment typical of nfvPPA in which both AOS and dysarthria often co-occur [4]. The severity and rapid progression of the motor speech impairment in FC were remarkable. She also showed progressive bucco-facial apraxia and some swallowing difficulties that worsened over time. The dissociation be-

Table 2
Voxel-based morphometry of grey matter changes in FC, comparing each year's scan against 17 female right-handed age-matched controls

| Demographic/functional | | | | | | | Controls mean (SD) |
|---|---|---|---|---|---|---|---|
| Age (screening visit, 2003) | 66 | | | | | | 69.5 (5.4) |
| Handedness (L/R) | R | | | | | | R |
| Education | 14 | | | | | | 16.3 (2.7) |
| Sex | F | | | | | | 5 M/5F |
| Age at disease onset | 63 | | | | | | – |
| **Follow-up visit (year)** | Year 1 | Year 2 | Year 3 | Year 4 | Year 5 | Year 6 | – |
| Years from first symptom | 3 | 4 | 5 | 6 | 7 | 8 | – |
| MMSE | 25 | 25 | 28 | 28 | 23 | 17 | 29.5 (0.7) |
| CDR | 0 | 0 | 0 | 0 | 0.5 | 0.5 | 0 |
| CDR sb | 0 | 0.5 | 0.5 | 0.5 | 0.5 | 1.5 | 0 |
| GDS | 3 | 2 | 3 | 2 | 8 | 11 | $\leqslant$ 10 |
| **Language production** | | | | | | | |
| Abbreviated Boston Naming test (15) | 9*** | 10*** | 11*** | 11*** | 6*** | 4*** | 14.4 (0.7) |
| Phonemic fluency (D words) | 5*** | 7*** | 9*** | 5*** | 3*** | – | 16.6 (6.8) |
| Semantic fluency (animals) | 9*** | 5*** | 5*** | 6*** | 5*** | | 21.2 (3.6) |
| Spontaneous speech fluency (WAB,10) | 2*** | 2*** | 1*** | 1*** | 0*** | 0*** | 10 (0) |
| Spontaneous speech information content (WAB, 10) | 8 | 2*** | 3*** | 2*** | 2*** | 0*** | 10 (0) |
| Repetition (WAB, 100) | 67* | 11** | 3*** | 0*** | 0*** | 0*** | 99.5 (0.9) |
| **Motor speech** | | | | | | | |
| Apraxia of speech rating (MSE), (7 = max deficit) | 6 | 7 | 7 | 7 | 7 | 7 | NA |
| Dysarthria rating (MSE, 7 = max deficit) | 2 | – | – | – | – | – | NA |
| **Verbal comprehension and semantics** | | | | | | | |
| Pyramid and Palm Trees Pictures (52) | 50* | – | 52 | 52 | 49** | 46** | 51.8 (0.4) |
| WAB Yes/No Comprehension (60) | 60 | 60 | 60 | 60 | – | 57 | 60 (0) |
| Auditory Word Recognition (WAB, 60) | 60 | 60 | 60 | 60 | 60 | 52* | 60 (0) |
| **Sentence comprehension** | | | | | | | |
| Sequential commands (WAB, 80) | 80 | 80 | 80 | 80 | 72* | 74* | 80 (0) |
| Syntactic comprehension (CYCLE, 55) | 52 | 48 | 49 | 48* | 47* | 44* | 53.8 (0.9) |
| **Visuo-spatial function** | | | | | | | |
| Benson Figure Copy (17) | 15 | – | 16 | 16 | 16 | 14 | 15.1 (1.7) |
| VOSP, number location (10) | 8* | 10 | 10 | 9 | 9 | 9 | $\geqslant$ 9 |
| Calculations (5) | 5 | 4 | 5 | 5 | 5 | 5 | $\geqslant$ 4 |
| **Episodic Memory** | | | | | | | |
| Benson Figure Delay (17) | 10 | 12 | 14 | 11 | 9 | 6* | 10.9 (3.9) |
| CVLT-MS 30-second free recall (9) | 8 | 7 | 8 | 7 | 6* | 3*** | 7.9 (1.6) |
| CVLT-MS 10-minute free recall (9) | 9 | 8 | 8 | 7 | 6* | 3*** | 7.3 (1.6) |
| **Executive function** | | | | | | | |
| Digit span backward | 3** | 3** | 4* | 4* | 3** | – | 4.9 (1.1) |
| Modified Trails no. lines/time(min) | 17.5** | 20.5* | 9.15** | 29.7* | 9.45** | 14** | 37.2 (9.8) |

BA: brain area; L: left; R: right; SFG: superior frontal gyrus; MFG: middle frontal gyrus; IFG p.o.: inferior frontal gyrus-pars opercularis; SMA: supplementary motor area; Prec.G: precentral gyrus; Postc. G.:postcentral gyrus; Amyg.: Amygdala; Hipp: hippocampus.

tween the severe motor speech impairment and the relative sparing of comprehension (even at the sentence level) in FC raises the question of whether she could be classified as "anarthric" or as having an "anterior opercular syndrome" as first described in the European literation of the 1990s" [36,37] and recently revived by Deramecourt [18]. Anarthric patients are thought to have pure motor speech impairment with dysarthria and bucco-facial apraxia with spared language comprehension and no agrammatism. Direct comparison of FC with less recent patients from the literature is difficult as formal language assessment was often limited and modern concepts such as AOS were often not applied. Nevertheless, we believe that at first presentation FC most closely resembled the "anarthric" rather than the "agrammatic" picture As disease progressed, FC showed clearer signs of grammatical impairment highlighting the fact that in many patients the boundaries between the anarthric and agrammatic forms can be ill-defined. For this reason, recent diagnostic criteria include both motor speech deficits and agrammatism as possible core symptoms necessary for nfvPPA diagnosis. Whether each of these two core features is predictive of specific anatomo-pathological substrates is under investigation [17,18,38]. It is worth noting that while many patients with nvfPPA and severe motor speech deficit evolve to develop a corticobasal syndrome [6,39], FC's non-speech motor deficits were never significant enough to warrant that syndromic diagnosis.

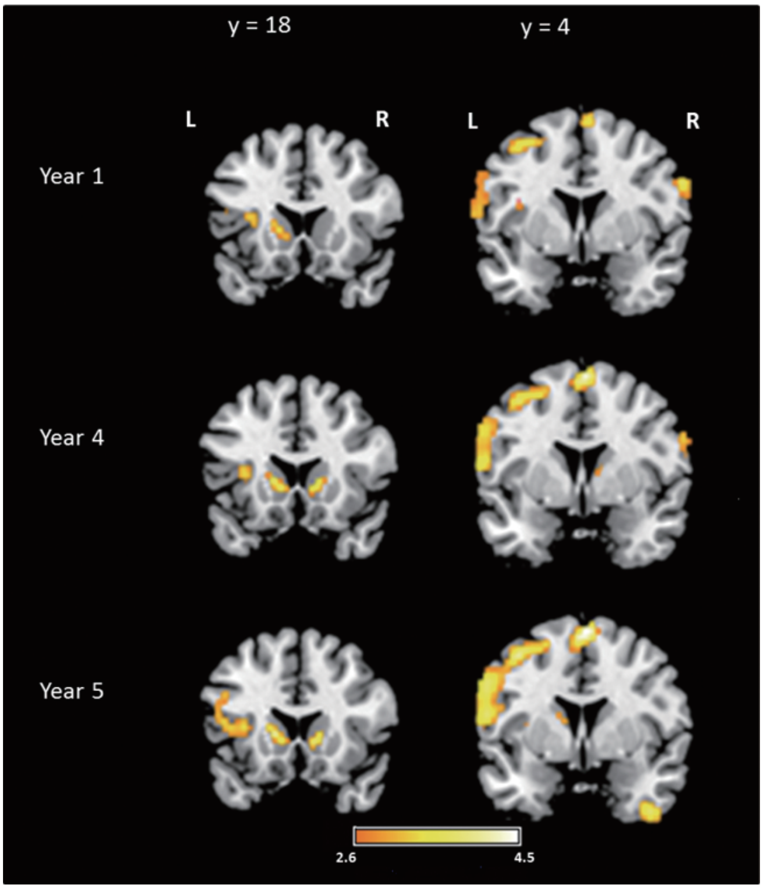

Fig. 2. VBM results of FC's scans performed in Year 1, Year 4 and Year 5 compared to controls. The patient underwent 5 scans (year 1-year 2-year 3- year 4- year 5). Since the same pattern of atrophy has been shown at year 1, 2 and 3, we present only year 1 MRI scan. Areas of atrophy indicated in yellow (see the color bar) are superimposed on coronal sections ($y = 18$; $y = 4$) of the mean image of all subjects used to create the template used for normalization. Include image statistical thresholding information. R: right; L: left.

Consistent with a diagnosis of nfvPPA with primarily motor speech impartment, FC's neuroimaging findings showed damage to the left frontal operculum/anterior insular region [6,37,40,41]. Interestingly, the left precentral regions corresponding to the face, mouth, and pharyngeal motor representations [42,43] were the most affected. Longitudinal VBM showed that atrophy became prominent also in other parts of the motor control network spreading to superior premotor cortex, SMA and basal ganglia as previously shown in nfvPPA [6,8]. Severe involvement of all these structures likely caused complete mutism in FC, stressing the concept that motor speech production is sustained by the interaction of multiple cortical and subcortical structures [44,45].

The prominence of the motor speech impairment in FC predicted an underlying tauopathy. While we predicted underlying CBD, the lack of general motor involvement even late in the course argued against this formulation. No clinical feature suggested AD pathology. In particular, FC's memory was spared until very late in the disease when all her cognitive functions declined. The posterior temporo-parietal regions typically involved in AD were not significantly atrophied on VBM or hypomethabolic on PET-FDG at year 5 (eight years from disease's onset). Hippocampal damage became apparent on VBM only five years after initial diagnosis (eight from first symptom). We were therefore surprised when the patient showed a PET-PiB positive scan. The literature on PET-PiB and FTD-spectrum clinical syndrome is still limited. The published data show that PiB positivity is in general infrequent in FTD and pathologically-confirmed patients that can elucidate the nature of the finding are rare [20]. In particular, FC is the first patient with nfvPPA to have pathologically confirmed FTLD despite a positive PiB-PET

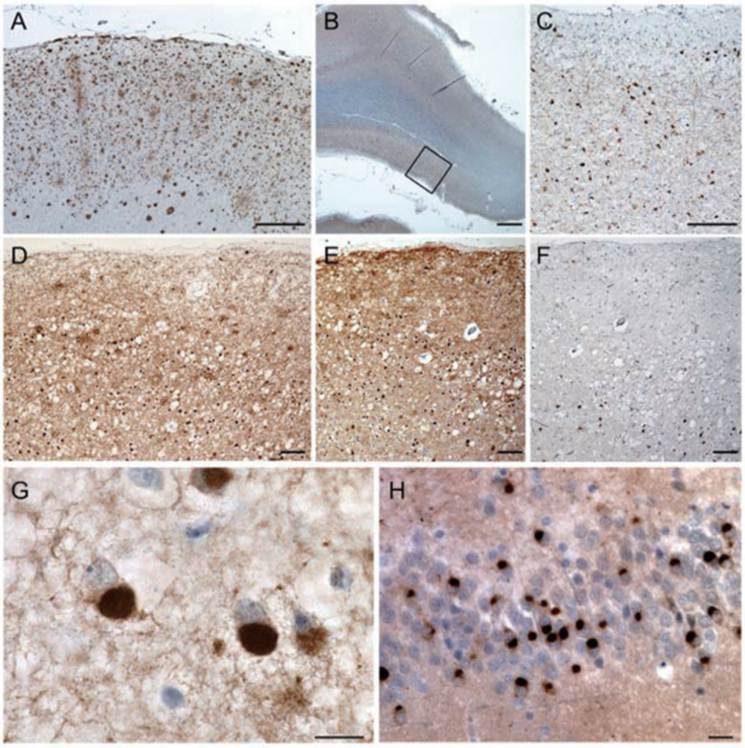

Fig. 3. Neuropathology. (A) Frequent diffuse and neuritic plaques, as well as moderate amyloid angiopathy, were seen in middle frontal gyrus and other neocortical regions using immunohistochemistry for amyloid-beta peptide. (B) Low magnification image of the precentral gyrus (extending into frontal operculum) reveals severe cortical thinning and abundant cortical tau pathology (CP-13 antibody to phosphorylated tau). (C) Magnified image of box in (B) reveals end-stage Pick's disease, with massive neuronal loss, dense neuropil threads, and scattered dystrophic astrocytes. (D) In mid-insula, a non-specific antibody to phosphorylated tau (CP-13) demonstrates Pick bodies and neuropil threads, as well as scattered neuritic plaques and neurofibrillary tangles. Immunohistochemistry to phosphorylated 3R (E) and 4R (F) tau confirms a predominantly 3R tauopathy, with only AD-related deep layer neurofibrillary tangles staining positively for 4-R tau. (G, H) Classical Pick bodies in mid-insula (G) and dentate gyrus (H) were identified with 3-R tau immunohistochemistry (G, H) but not with a 4-R tau antibody or Gallyas silver staining (not shown). Scale bars = 500 $\mu$M (A), 1 mm (B), 500 $\mu$M (C), 100 $\mu$M (D-F), 10 $\mu$M (G), and 25 $\mu$M (H).

scan. She was also included in a previous series from our group [22]. Recently, we encountered another patient with PIB positive nfvPPA, but the patient showed a more mixed clinical picture and remains alive at the time of this writing [20]. Another clinical series was recently reported in which only two of eight nfvPPA patients were found to be positive [21]. Concerning our patient, many possible ante-mortem explanations could be hypothesized for her PiB positivity. Though FC filled all the criteria for definite nfvPPA, PiB-PET positivity could suggest an atypical presentation of AD. In PPA, the logopenic-variant (lvPPA) has been most often associated with PIB+ and AD pathology [2,46] but in our patient both clinical and MRI/FDG-PET data excluded an lvPPA diagnosis. On the other hand, few patients with nfvPPA and underlying AD pathology have been described. High frequency of AD pathology has been reported in language-impaired patients with clinical syndromes classified as having a "nonfluent" presentation that are sometimes, but not always, consistent with lvPPA [12,14,47].

Grossmann also followed nine patients with nfvPPA longitudinally, and noted AD pathology in three patients at autopsy [48]. We might also interpret FC's PiB positivity as a an incidental finding, since up to a third of cognitively normal elderly population shows positive PiB-PET scan [49,50] or as a preclinical phase of AD, taking into account that cognitively normal individuals with positive PiB-PET scan have a greater risk of progression to symptomatic AD after 3–4 years of follow up [51]. PIB positivity would therefore not exclude FTLD pathology as the cause of FC's aphasia syndrome but might instead highlight an overlapping amyloid pathology that in our patient appears to have been "clinically silent" until later stages of the disease. The clinical course of FC's disease was characterized by a general cognitive decline only in the last years of disease. Post-mortem pathological findings of Pick's disease with AD co-pathology (Braak stage V) further support the latter hypothesis.

To our knowledge, this is the first prospective, longitudinal description of nfvPPA patient in which in-vivo evidence of brain amyloidosis detected with PiB-PET scan was correlated with pathological diagnosis in the same patient. The principal pathological diagnosis in our patient was Pick's disease. We conclude that in-vivo biomarker evidence of brain amyloidosis (PiB-PET scan) should not be considered conclusive evidence that AD is responsible for a typical FTD syndrome.

## Acknowledgments

We thank patients and their families for the time and effort they dedicated to our research.

We also thank William Jagust for PET scanning.

The study was supported by grants from the National Institutes of Health (NINDS R01 NS050915, NIA P50 AG03006, NIA P50 AG023501, NIA P01 AG019724); State of California (DHS04-35516); Alzheimer's Disease Research Centre of California (03-75271 DHS/ ADP/ARCC); Larry L. Hillblom Foundation; John Douglas French Alzheimer's Foundation; Koret Family Foundation; Consortium for Frontotemporal Dementia Research; and McBean Family Foundation.
